# Presynaptic protein Synaptotagmin1 regulates the neuronal polarity and axon differentiation in cultured hippocampal neurons

**DOI:** 10.1186/s12868-015-0231-x

**Published:** 2015-12-15

**Authors:** Yuriko Inoue, Yuji Kamikubo, Hiromitsu Ezure, Junji Ito, Yu Kato, Hiroshi Moriyama, Naruhito Otsuka

**Affiliations:** Department of Anatomy, Showa University School of Medicine, Tokyo, 142-8555 Japan; Department of Pharmacology, Juntendo University School of Medicine, Tokyo, 113-8421 Japan; School of Nursing and Rehabilitation Sciences, Showa University Department of Nursing, Tokyo, 226-8555 Japan; Department of Neurosurgery, Showa University School of Medicine, Tokyo, 142-8555 Japan

**Keywords:** Synaptotagmin1, Axon, Dendrite, Tau1, NaCh, shRNA

## Abstract

**Background:**

Hippocampal neurons in the brain polarize to form multiple dendrites and one long axon. The formation of central synapses remains poorly understood. Although several of the intracellular proteins involved in the clustering of central neurotransmitter receptors and ion channels have been identified, the signals involved in pre- and postsynaptic differentiation remain elusive. Synaptotagmin1 is an abundant and important presynaptic vesicle protein that binds Ca^2+^ (J Biol Chem 277:7629–7632, 2002) in regulation of synaptic vesicle exocytosis at the synapse. Synapse consists of the formation of synaptic connections and requires precise coordination of Synaptotagmin1. It was reported Synaptotagmin1 plays an important roles in the formation of axonal filopodia and branches in chicken forebrain neurons (Dev Neurobiol 73:27–44, 2013). To determine if Synaptotagmin1 could have a role in formation of axon in hippocampal neurons, we investigated the effects of Synaptotagmin1 overexpression and knockdown using the shRNA on the growth and branching of the axons of primary hippocampal neurons. We showed that overexpression of Synaptotagmin1 leads to abnormal multiple axon formation in cultured rat hippocampal neurons.

**Results:**

We first examined the effects of Synaptotagmin1 on the numbers of axon and dendrites. We found that the overexpression of Synaptotagmin1 led to the formation of multiple axons and induced an increase in the number of endogenous postsynaptic protein Homer1c clusters in cultured hippocampal neurons. Endogenous initial segment of axon was detected with anti-sodium channel (anti-NaCh) antibody and with anti-Tau1 (J Neurosci 24: 4605–4613, 2004). The endogenous initial segment of axon was stained with anti-NaCh antibodies and with anti-Tau1 antibodies. Then the numbers of prominence dyed positive were counted as axon. We attempted to specifically knockdown the endogenous Synaptotagmin1 with small hairpin RNAs (shRNAs). To further dissect the functions of endogenous Synaptotagmin1 in neuronal polarity, we used the shRNA of Synaptotagmin1 that specifically blocks the existence of endogenous Synaptotagmin1. When the shRNA of Synaptotagmin1 was introduced to the cells, the number of axons and dendrites did not change.

**Conclusions:**

These results indicate that the accumulation of Synaptotagmin1 may play an important role in axon/dendrite differentiation.

## Background

The formation of central synapses remains poorly understood. Although several of the intracellular proteins involved in the clustering of central neurotransmitter receptors and ion channels have been identified, the signals involved in pre- and postsynaptic differentiation remain elusive.

Ca^2+^ influx into presynaptic nerve terminals activates synaptic vesicle exocytose by triggering fast synchronous fusion and a slower asynchronous release pathway. In addition, a brief rise in Ca^2+^ after consecutive action potentials has been correlated with a form of short-term synaptic plasticity with enhanced vesicle fusion termed facilitation. Synaptotagmin1 was originally identified as an abundant synaptic vesicle protein that binds Ca^2+^ and phospholipids. It is now widely thought to be the major Ca^2+^ sensor for neurotransmitter release from lower invertebrates to mammals [1, 4]. Its role is currently unclear in relation to neuronal development such as axon differentiation in hippocampal neurons. Previously, it was reported that the activity-dependent re-organization of central synapses is thought to play an important role in learning and memory [5, 6]. It is characterized by the coordinated regulation of pre- (axons) and postsynaptic sites (dendrites). Most of the excitatory synapses are located on dendritic spines. Neuronal activity induces a variety of changes in spine morphology and the distribution of postsynaptic proteins in spines [7–11].

Axon/dendrite differentiation is a critical step in neuronal development. Hippocampal neurons have only one axon formation. Neuronal polarization may involve an initial specification of axon/dendrite identity in undifferentiated neuritis, followed by the selective trafficking and segregation of components into the axon and the dendrites [12, 13]. Many important presynaptic proteins, such as Synaptotagmin1, organize a single axon and it is possible that these proteins are related to the neuronal polarity. It was reported Synaptotagmin1 plays an important roles in the formation of axonal filopodia and branches in chicken forebrain neurons [2]. Dissociated hippocampal neurons in culture have been widely used to study neuronal polarization [14]. Determining how a neuron acquires its polarity is a fascinating question that is under intensive study.

In this study, we attempted to determine significance of Synaptotagmin1 proteins in hippocampal neurons. For this purpose, we exogenously overexpressed Synaptotagmin1 (a gift from Dr. Thomas C Sudhof) [15, 16].

To determine if Synaptotagmin1 could have a role in formation of axon in hippocampal neurons, we investigated the effects of Synaptotagmin1 overexpression and knockdown using the shRNA on the growth and branching of the axons of primary hippocampal neurons. Overexpression of Synaptotagmin1 leads to abnormal multiple axon formation in cultured rat hippocampal neurons.

## Methods

### Cell cultures

The primary cultures of hippocampal neurons were prepared as described previously [17]. Briefly, hippocampi were isolated from Wistar rats at 18 days of gestation (E18), and treated with papain (100 mg/ml, Worthington, Lakewood, NJ, USA) for 10 min at 37 °C. Dissociated neurons were plated at a density of 20,000–30,000 cells/cm^2^ onto cover glasses (Matsunami, Osaka, Japan) that were coated with 1 mg/ml poly-l-lysine (Sigma), cultured in Neurobasal-A medium (Invitrogen) supplemented with 2 % B-27 (Invitrogen) and 0.5 μM glutamine, in a humidified atmosphere of 5 % CO_2_ at 37 °C for 14–21 days. Half of the medium was exchanged every other week. All experiments conformed to the guidelines of the Showa University Animal Welfare Committee on the ethical use of animals. Every efforts were made to minimize the number of animals used and their suffering.

### Constructions of plasmid DNAs

The cDNAs encoding Synaptotagmin1 were cloned in frame at EcoRI and BamHI sites in pVenus-C1 (a gift from Dr. Miyawaki). Venus is a variant of yellow fluorescent protein (YFP) that features improved brightness. Purified plasmid DNAs (1 μg/μl) were overexpressed into the nuclei of pyramidal neurons at 14–19 days in vitro using Lipofectamin2000 (Invitrogen).

We selected the target sequences for the rat Synaptotagmin1-specific shRNA as Fukuda et al. described previously [18]. The target sequences were chosen within the region specific to rat Synaptotagmin1and sequences do not match with any other rat genes. We designed Synaptotagmin1 shRNA as follows: 5′-GCTGAAGCAGAAGTTTATG-3′. Plasmids were constructed using the pAVU6+27 vector with U6 RNA polymerase III promoter (a gift from Dr. David R. Engelke) [19]. A scrambled sequence of Synaptotagmin1 shRNA (5′-TCGGAACAGGATTATTGA-3′) was used as a negative control.

The endogenous initial segment of axon was stained with anti-sodium channel (NaCh) rabbit antibody (1:100 dilutions, Jackson Immuno Research) and with anti-Tau1 (1:100 dilutions, Chemicon) [3]. Then the numbers of axon were counted. The numbers of dendrites were counted from a fluorescent images of Venus or by staining of MAP2 antibody (1:100 dilutions, Chemicon), which appeared to remain healthy by observing the imaging of neuronal dendrites morphology. Synaptotagmin1 shRNA and scrambled Synaptotagmin1 shRNA (1 μg/μl in PBS) together with Venus was transfected into neurons [18]. Successful transfections were confirmed by Venus yellow fluorescence. As controls, we used the same concentration of scrambled Synaptotagmin1 shRNA.

### Immunocytochemistry

We used HEK293T cells but not hippocampal primary cultures to investigate the knockdown efficiency of Synaptotagmin1 shRNA in Immunocytochemistry because HEK293T cells have transfection efficiency higher than 1000 or more times than those of neurons. Transfections of HEK293T cells were performed with 1 μg of Venus-Synaptotagmin1 DNA by lipofection using Lipofectamine2000 (Life Technologies) according to the manufacturer’s instructions. After 36 h of expression, these cells were used for live imaging, or were fixed for immunocytochemistry. Cultured neurons and HEK293T cells were fixed with 4 % paraformaldehyde in phosphate-buffered saline (PBS) for 30 min. Cells were permeabilized with 0.2 % Triton X-100 in PBS for 5 min and incubated with 8 % BSA in PBS for 30 min. The antibodies used were rabbit anti-Homer1c (1:400) [20] and mouse anti-Synaptotagmin (1:200, Chemicon).

### Immunoblots of transiently expressed proteins in HEK293T cells

HEK293T cells were transfected with Venus-Synaptotagmin1 by lipofection using Lipofectamine2000 (Life Technologies) according to the manufacturer’s instructions. After 36 h, proteins were extracted in 2× sodium dodecyl sulfate sample buffer. Equal amounts of cell extracts were analyzed by western blotting using anti-Synaptotagmin1 or anti-tubulin antibody as a control. To measure the expression levels of Synaptotagmin1 and tubulin, polyvinylidene difluoride (PVDF) membranes were fixed for 45 min with 4 % PFA in PBS at 4 °C and rinsed three times with PBS for 20 min before blocking [21].

### Image analysis

Specimens were examined using an LSM510 laser scanning confocal microscope (Carl Zeiss, Germany). Confocal images were obtained using a 40× objective lens at 1024 × 1024 pixel resolution. The laser intensity and gain were adjusted to avoid saturation of the maximum pixel intensity and photobleaching. Neurons of pyramidal shape were morphologically identified. Segments of 75 μm (from 5 to 80 μm distal from the soma) of major dendrites were examined. Neurons of pyramidal shape were morphologically identified. Once set and optimized, individual samples were imaged at the same optical settings.

Images were projected in z-series consisting of three to five slices at 1–1.5 μm intervals, covering the entire z-plane of cultured neurons. Double-labeled neurons were chosen randomly from two to five coverslips. Each experiment was repeated at least three times. The number of neurons used for analyses is indicated in figure legends. Morphometric measurements were carried out with Image Browser (Carl Zeiss, Germany). Immunoreactive clusters of synaptic proteins on dendritic spines and shafts were defined as spots of 0.5–2 μm in diameter over 50 % of the maximum pixel intensity of examined areas unless otherwise specified. We defined dendritic spines as protrusions with an enlarged tip of less than 4 μm in length and narrower than 1 μm in spine neck on dendrites.

Endogenous initial segment of axon was detected with anti-NaCh antibody (1:100 dilutions, Jackson Immuno Research) and with anti-Tau1 [3]. Then the numbers of axon were counted by staining with anti-NaCh antibodies and with anti-Tau1 antibodies.

## Results

### Effect of morphology of neuron after over expression of Synaptotagmin1

**Fig. 1:**
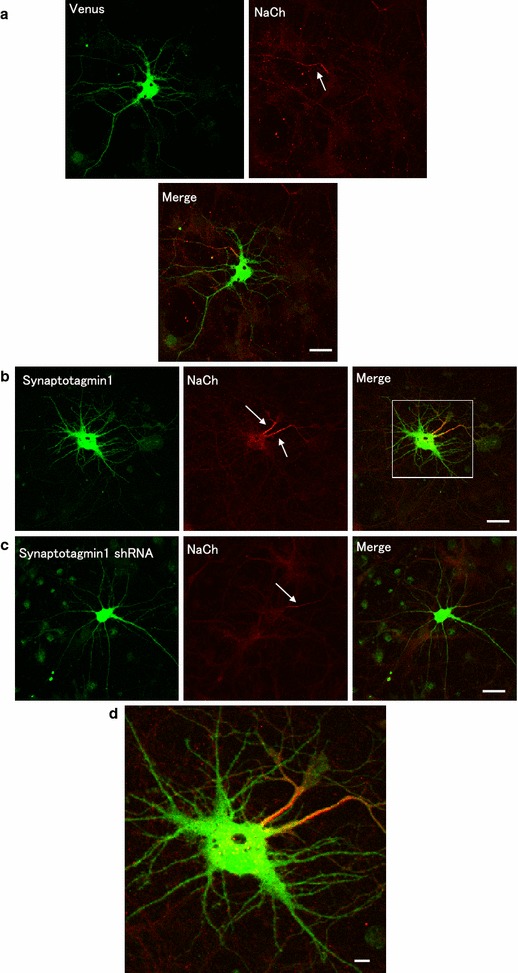
Distribution of synaptic proteins in hippocampal neurons. **a** Fluorescent images of a hippocampal neuron expressing Venus (*top left panel*), endogenous-NaCh (*top right panel*) and merged panel (*down panel*). **b** Fluorescent images of a hippocampal neuron expressing Venus-Synaptotagmin1 (*left panel*), endogenous-NaCh (*middle panel*) and merged panel (*right panel*). **c** Venus + Synaptotagmin1 shRNA, endogenous-NaCh (*middle panel*) and merged panel (*right panel*). **d** This is the figure which expanded covered by the *white frame*. *Arrows* show the axon. *Scale bars* = 10 μm

**Fig. 2:**
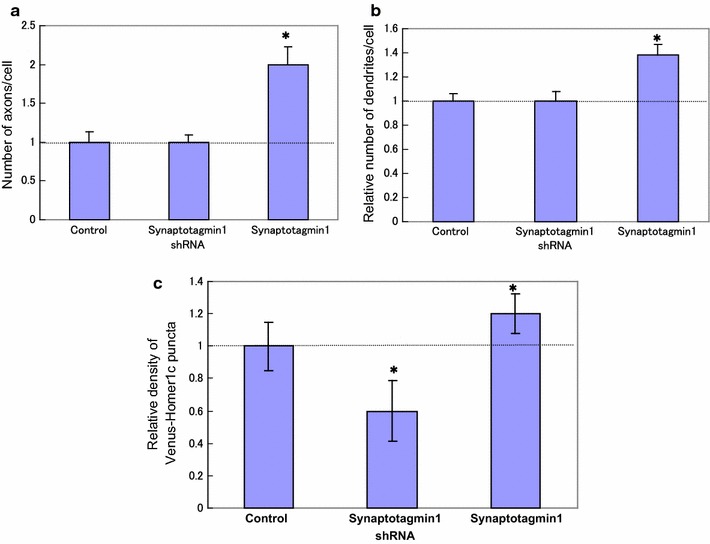
**a** Relative number of axons/cell in only Venus plasmids (*control left*), Venus-Synaptotagmin1 shRNA (*middle*), and Venus-Synaptotagmin1 (*right*). **b** Relative number of dendrites/cell Venus-Control (*left*), Venus + Synaptotagmin1 shRNA (*middle*), and Venus-Synaptotagmin1 (*right*). **c** Relative number of Venus Homer1c puncta after transfection of Synaptotagmin1. Only Venus-plasmids (*control left*), Venus-+Synaptotagmin1 shRNA (*middle*), and Venus-Synaptotagmin1 (*right*). The cluster density of these proteins at time 0 was 1, 0.62, and 1.23 clusters per micrometer, respectively. Error bars represent ± standard error of the mean (SEM) (n = 40 dendrites). *Error bars* represent ± SEM (n = 10 neurons). *p < 0.05 and NS (no significant; *p* > 0.15) by Student’s *t* test

**Fig. 3:**
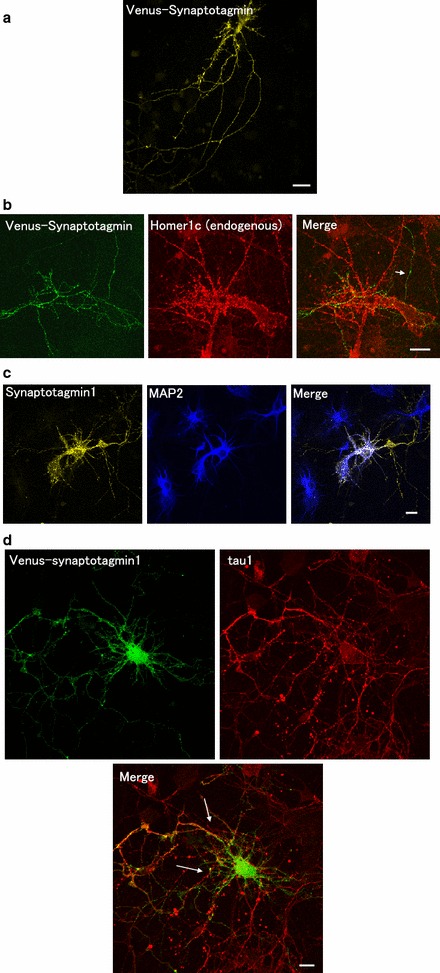
Fluorescent images of a hippocampal neuron expressing Venus-Synaptotagmin1. **a** Fluorescent images of Venus-Synaptotagmin1 in hippocampal neuron. **b** Double staining of Venus-Synaptotagmin1 (*left panel*) and endogenous-Homer1c (*middle panel*). **c** Double staining of Venus-Synaptotagmin1 (*left panel*) and endogenous-MAP2 (*middle panel*). **d** Double staining of Venus-Synaptotagmin1 (*left panel*) and endogenous-Tau1 (*right panel*). *Arrows* show the axon. *Scale bars* = 10 μm

We showed that hippocampal neurons expressed Venus in the brain polarize to form multiple dendrites and one long axon (Fig. 1a). As shown in Figs. 1b and 2b, we found that the overexpression of Venus-Synaptotagmin1 led to the formation of multiple dendrites. The number of axons and dendrites significantly increased in neurons expressing Venus-Synaptotagmin1 (Figs. 1b, d, 2, 3c respectively) compared to that in neurons expressing Venus-Synaptotagmin1 shRNA (Figs. 1c, 2). Endogenous postsynaptic protein Homer1c exists in dendrites and does not exist in axon in cultured hippocampal neurons (Fig. 3b).

The endogenous initial segment of the axon was detected with a specific antigen of axon anti-sodium channel (NaCh) (Fig. 1) and with anti-Tau1 (Fig. 3d). Next, we observed that many endogenous initial segments of axons were stained with anti-Tau1 (Fig. 3d). Morphological examination of the neurons revealed that Venus-Synaptotagmin1 was highly abnormal and that Tau-1 staining was abnormally strong, suggesting that there were multiple axons (Figs. 1, 2 and 3d). The number of dendrites was then counted (Figs. 1, 2b and 3c). We found that the overexpression of Synaptotagmin1 led to multiple axon and dendrites formation in cultured hippocampal neurons. Cultured neurons were transfected with Venus-Synaptotagmin1 shRNA (control) or Venus-Synaptotagmin1 DNA. The number of axon and dendrites was both significantly increased in neurons expressing Venus-Synaptotagmin1 compared to that in neurons expressing Venus (control), scrambled shRNA (negative control) and Synaptotagmin1shRNA (Figs. 1, 2a, b).

### Confirmation of construction of Synaptotagmin1 shRNAs to knockdown the Synaptotagmin1 expression

**Fig. 4:**
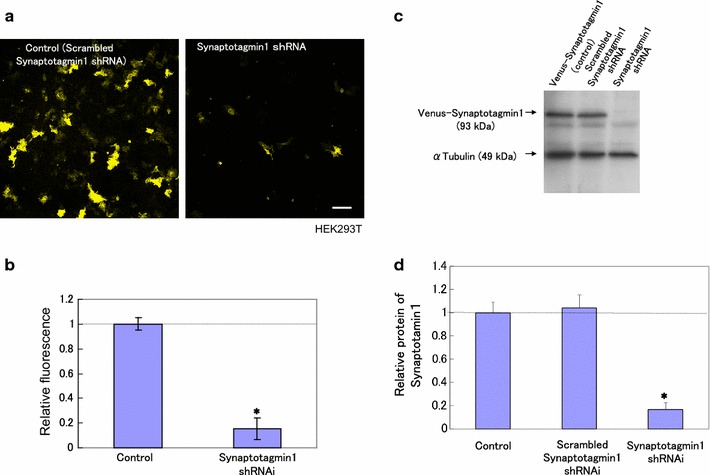
Construction of Synaptotagmin1 shRNA. **a** Fluorescent images of Venus-Synaptotagmin1 in HEK293T cells (*left*) and Venus-Synaptotagmin1 and Synaptotagmin1 shRNA in HEK293T cells (*right*). *Scale bars* = 10 μm. **b** The *graph* shows the relative fluorescence of Synaptitagmin1 cells after transfection of shRNA constructs. *p < 0.005 by Student’s *t* test against the control. **c**, **d** Western blot analysis of transfected HEK293T. Cells were transfected with Venus-Synaptotagmin1

To further understand the role of Synaptotagmin1 in neurons, we attempted to specifically knockdown the endogenous Synaptotagmin1 with small hairpin RNAs (shRNAs) (Fig. 4a–d).

We designed four shRNAs targeting the untranslated region (UTR) of rat Synaptotagmin1 mRNA [18]. The effectiveness and specificity of these Synaptotagmin1 shRNAs were first examined in HEK293T cells.

Venus-Synaptotagmin1 was used as an indicator, which was diffusedly expressed in the cytoplasm of HEK293T cells. When the shRNAs were co-transfected with Venus-Synaptotagmin1 to the HEK293T cells, Synaptotagmin1 shRNA was found to most significantly down-regulate the expression of Venus-Synaptotagmin1 plasmids, without affecting the level of a cellular protein, tubulin (Fig. 4a–d). Furthermore, a scrambled shRNA (negative control) did not affect the level of Venus plasmids expression (data not shown). We also analyzed the level and specificity of Synaptotagmin1 down-regulation by western blot analyses (Fig. 4a–d). Synaptotagmin1 shRNA, but not scrambled shRNA, significantly reduced the expression of Venus-Synaptotagmin1 (93 kD), whereas the expression of tubulin was not affected.

### Overexpression of Synaptotagmin1 greatly affects the morphology of neurons

We transected with the Venus-Synaptotagmin1 and observed the morphology of neurons. Venus-Synaptotagmin1 significantly affected the morphology of neurons; for example, there were many axons and neuronal polarity with double staining of MAP2. MAP2 is a microtubule protein and is used as a cytoskeleton (Fig. 3c). Importantly, we showed that Synaptotagmin1 affect the polarity of hippocampal neurons.

**Fig. 5:**
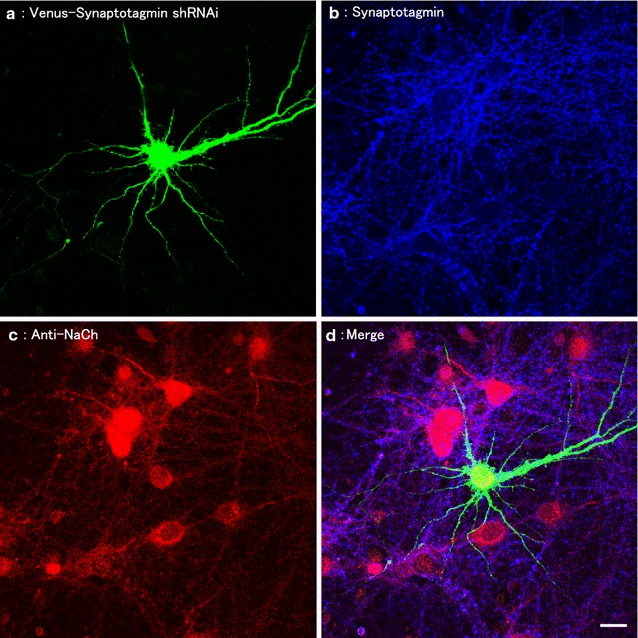
Fluorescent images of hippocampal neurons expressing Synaptotagmin1 shRNA. **a** Triple staining of Venus-Synaptotagmin1 (*top left panel*), **b** endogenous-Synaptotagmin1 (*top right panel*), **c** endogenous-Tau1 (*down left panel*) and **d** merged panel (*down right panel*). Fluorescent images of hippocampal neurons expressing Synaptotagmin1 shRNA after transfection. *Scale bar* = 10 μm

Next, we examined the effectiveness of Synaptotagmin1 shRNA in cultured hippocampal neurons (Fig. 5). We showed that Synaptotagmin1 shRNA induced specifically knockdown the endogenous Synaptotagmin1. Synaptotagmin1 shRNA specifically blocks the expression of endogenous Synaptotagmin1 but not endogenous Homer1c and anti-NaCh in hippocampal neurons. In summary, our dates indicate that Synaptotagmin1 is important for the control of the neuronal polarity.

## Discussion

The formation of a multi synaptic glomerular rosette requires interactions between a mossy fiber and several GCs. Here, we demonstrate that Synaptotagmin1, secreted by GCs, plays a role in this process.

Many signaling molecules, such as upstream regulators PI3K and PTEN, were found to be essential for neuronal polarization. They were activated by PI3K and were located at an upstream position in the signaling pathways for the neuronal polarization involved in many molecules, such as the axon-specific microtubule-associated protein CRMP-2 [22], the mammalian partitioning-defective (PAR) proteins PAR-3 [23, 24], PAR-6 [24], the small GTPases Rap1B [25], Cdc42 [26, 27], GSK-3β [22], the plus-end motor proteins KIF3A [23], KIF5C [28], MARK2 [29], the insulin-like growth factor-1 [28], and a neuron-specific protein Shootin1 [30]. Overexpression of these proteins is known to lead to multiple axon formation and dendrites.

Previously, it was reported that Synaptotagmin1 interacted with PtdInsP_3_ kinase. PtdIns (3, 4 and 5) P_3_ is known to activate PI3K directly. Overexpression of Synaptotagmin1 may activate PtdIns (3, 4 and 5) P_3_ and then trigger the activation of PI3K and these downstream proteins. Activation of these proteins may cause multiple axon formation. It was reported that the polarity in hippocampal neurons is generated in the initial 2–3 days after platting [31, 32] cv. In this study, we showed the neuronal polarity in 2 weeks after platting. In summary, these data suggest that Synaptotagmin1 is required (at least in part) for axon specification and neuronal polarity for an extended period.

We previously reported biphasic changes in the distribution of Venus-Homer1c clusters after glutamate stimulation in living neurons [33]. Previously, fluorescent images of the dendrites of a cell expressing Venus-Homer1c taken at various time points after glutamate stimulation (100 μM, 1 min). Since there is a possibility that the reduction of clusters may represent the loss of synapses, we counted the number of dendritic spines in neurons expressing Venus only. In previous report, the glutamate stimulation caused a monotonic and gradual increase in the number of spines without the initial reduction observed for postsynaptic proteins [33]. These results suggest that the glutamate stimulation initially induces the clustering of postsynaptic proteins at pre-existing spines and then leads to the reassembly of postsynaptic proteins along with the formation of new synapses. When the localization of synaptotagmin1, a typical presynaptic marker, was similarly examined, most of the synaptotagmin1 clusters were found to be juxtaposed with those of postsynaptic proteins (Homer1c). These results indicate that Homer1c and Synaptotagmin1 exhibited the main synaptic localization.

These results suggest that the time courses of Synaptotagmin1 induction and the initial disassembly of postsynaptic proteins are well correlated and that the induction of Synaptotagmin1 may have contributed to the reorganization of postsynaptic structures.

## Abbreviations

DIV: days in vitro
NaCh: voltage-gated sodium channel
F-actin: Filamentous actin
PBS: phosphate buffered saline
shRNA: short hairpin RNA
